# Differential impact of water immersion on arterial blood flow and shear stress in the carotid and brachial arteries of humans

**DOI:** 10.14814/phy2.13285

**Published:** 2017-05-30

**Authors:** Howard H. Carter, Angela L. Spence, Philip N. Ainslie, Christopher J. A. Pugh, Louise H. Naylor, Daniel J. Green

**Affiliations:** ^1^ School of Human Sciences The University of Western Australia Crawley Western Australia; ^2^ Centre for Heart, Lung and Vascular Health University of British Columbia, Okanagan Campus School of Health and Exercise Sciences Kelowna British Columbia Canada; ^3^ Cardiff School of Sport Cardiff Metropolitan University Cardiff United Kingdom; ^4^ Research Institute for Sport and Exercise Science Liverpool John Moore's University Liverpool United Kingdom

**Keywords:** Arteries, mean arterial pressure, shear stress, water immersion

## Abstract

Arterial shear stress is a potent stimulus to vascular adaptation in humans. Typically, increases in retrograde shear have been found to acutely impair vascular function while increases in antegrade shear enhance function. We hypothesized that blood flow and shear stress through the brachial and carotid arteries would change in a similar manner in response to water immersion, an intervention which modifies hemodynamics. Nine healthy young male subjects were recruited to undergo controlled water immersion in a standing upright position to the level of the right atrium in 30°C water. Diameters were continuously and simultaneously recorded in the brachial and common carotid arteries along with mean arterial pressure (MAP), cardiac output (CO), and heart rate before, during, and after 10 min of immersion. MAP and CO increased during water immersion (baseline vs. 8–10 min; 80 ± 9 vs. 91 ± 12 mmHg; and 4.8 ± 0.7 vs. 5.1 ± 0.6 L/min, *P* < 0.01 and *P* < 0.05, respectively). We observed a differential regulation of flow and shear stress patterns in the brachial and carotid arteries in response to water immersion; brachial conductance decreased markedly in response to immersion (1.25 ± 0.56 vs. 0.57 ± 0.30 mL.min/mmHg, *P* < 0.05), whereas it was unaltered in the carotid artery (5.82 ± 2.14 vs. 5.60 ± 1.59). Our findings indicate that adaptations to systemic stimuli and arterial adaptation may be vessel bed specific in humans, highlighting the need to assess multiple vascular sites in future studies.

## Introduction

It is now well established that repeated episodic increases in blood flow and shear stress are important stimuli for arterial adaptation (Hambrecht et al. 2003; Tinken et al. 2010) and health (Pohl et al. 1986; Rubanyi et al. 1986; Joyner and Green 2009). Recent advances in imaging and analysis have revealed that blood flow through arteries in humans is not always unidirectional and that diastolic retrograde flow occurs under some circumstances (Green et al. 2002). This led to studies characterizing the distinct impacts of different flow and shear patterns on artery function (Thijssen et al. 2009a, 2009b; Tinken et al. 2009; Carter et al. 2013); while increases in systolic anterograde flow tend to enhance endothelium‐dependent vasodilator function, unopposed increases in retrograde flow are associated with acute endothelial injury (Jenkins et al. 2013) and impaired flow‐mediated dilation (Thijssen et al. 2009b; Newcomer et al. 2011). Few studies, however, have simultaneously assessed changes in flow and shear patterns in different vascular beds in vivo*,* which is important when attempting to determine the overall impact of a modality on vascular function and health. In this study, we took advantage of recent observations that water immersion alters systemic hemodynamics at rest in humans (Carter et al. 2014) to manipulate mean arterial pressure (MAP) and simultaneously measure the magnitude and pattern of blood flow and shear through the common carotid and brachial arteries in vivo. We examined the hypothesis that water immersion‐induced changes in systemic hemodynamics would result in similar changes in artery blood flow and shear rate in both vascular territories.

## Materials and Methods

### Ethical approval

This study complied with the *Declaration of Helsinki* and the Human Research Ethics Committee of the University of Western Australia approved the experimental protocols. All subjects provided written, informed consent before participating in the study.

### Subject characteristics

Nine young and healthy recreationally active men, performing ≤2 h of physical activity per week, were recruited (24.6 ± 2.0 years). The participant's average height and weight were 1.74 ± 0.09 m and 76 ± 9 kg, respectively, with an average BMI of 25.0 ± 1.7 kg.m^−2^. Exclusion criteria included any history of cardiovascular, musculoskeletal or metabolic disease, smoking, and individuals on medication. Women were excluded from this study due to the cyclical effects of estrogen on hemodynamic and vascular variables.

## Experimental procedures

Subjects arrived at the laboratory having fasted for a minimum of 8 h and abstained from alcohol, caffeine, and vigorous exercise for at least 24 h. Upon arrival, subjects were seated and instrumented (~30 min). They were then positioned in an empty tank (1.4 m diameter, 1.55 m height, 2400L) in a standing position with their arms resting comfortably on a platform out of the water at heart level. Subjects were asked to remain as stationary as possible throughout the experiment to avoid excessive movement. This experimental approach avoided the potential for confounding effects of movement into, or out of, the tank on hemodynamics. Following a 10 min baseline period of quiet rest, three submersible water pumps (KPA 600A; Grundfos, South Australia) filled the tank at a constant rate with 30°C water to the level of the right atrium (RA), a process that was completed in 7 min. The subjects remained immersed at the level of the RA for 10 min while remaining in a stationary standing posture, after which time the pumps were reversed and water rapidly evacuated at a constant rate. Subjects attended the laboratory wearing shorts and a t‐shirt. The average ambient air temperature throughout the testing sessions was 26.0 ± 3.4°C. Prior to the commencement of baseline recording, following instrumentation, t‐shirts were removed for the duration of the testing session. Data were measured and recorded continuously throughout the entire protocol. The common carotid artery diameter and blood pressure data presented has been previously published (Carter et al. 2014).

## Experimental measures

### Assessment of common carotid and brachial artery diameter and velocity

Following the 10 min baseline period, common carotid and brachial artery diameter and velocities were simultaneously recorded throughout the protocol using a 10‐MHz multifrequency linear array probe attached to a high‐resolution ultrasound machine (T3000; Terason, Burlington, MA). Recording began following optimization of the longitudinal B‐mode image of the lumen‐arterial walls. Concurrently, Doppler velocity assessments were collected using the lowest possible insonation angle (always <60°). The subject's arms were supported at heart level throughout the duration of the test.

Analysis of artery diameter and flow was performed using custom‐designed edge‐detection and wall‐tracking software, which is independent of investigator bias and has previously been comprehensively described (Woodman et al. 2001; Black et al. 2008). From synchronized diameter and velocity data, blood flow (the product of lumen cross‐sectional area and Doppler velocity) was calculated at 30 Hz. Shear rate, an estimate of shear stress without viscosity, was calculated as 4× mean blood velocity/vessel diameter. Reproducibility of diameter measurements using this semiautomated software is significantly better than manual methods, reduces observer error and bias significantly, and possesses an intraobserver coefficient of variance of 6.7% (Woodman et al. 2001).

### Systemic hemodynamics

A Finometer PRO (Finapres Medical Systems, Netherlands) was used to measure changes in MAP, heart rate (HR), cardiac output (CO), and stroke volume (SV) via photoplethysmography. These data were exported to a data acquisition system PowerLab (LabChart 7, ADInstruments, Sydney, Australia) in real time. The finger cuff was placed around the middle finger of an arm supported at RA level on a platform. The subject was instructed not to move their arm or finger during recording. All data were averaged across 1–2 min.

## Statistics

Statistical analysis was performed using SPSS 19.0 (SPSS, Chicago, Illinois) software. Repeat‐measure ANOVAs were performed and any significant values were followed up with post hoc *t*‐tests. Statistical significance was assumed at *P* < 0.05. All data are reported as mean ± SD unless stated otherwise.

## Results

### Common carotid and brachial artery responses

#### Blood flow

**Figure 1:**
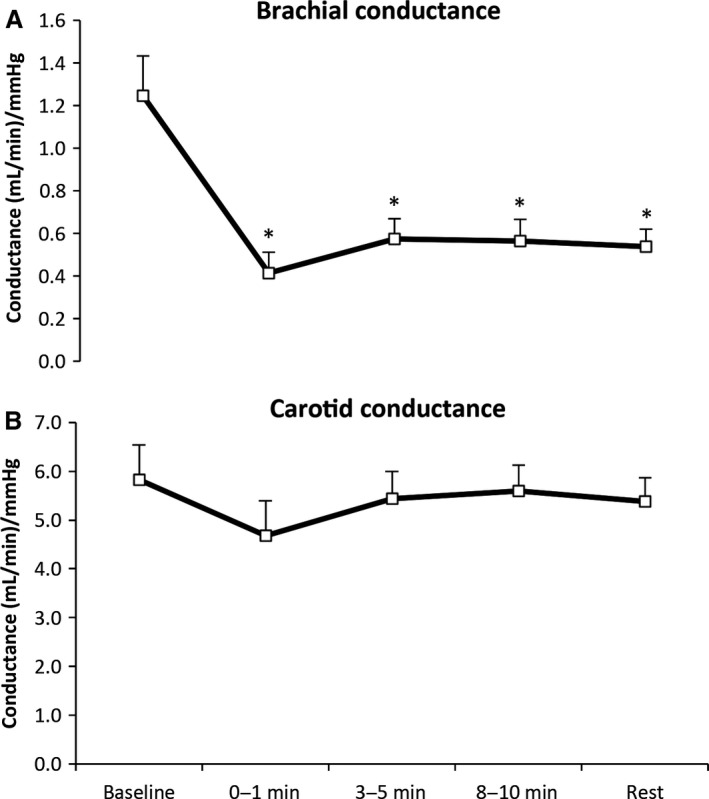
Brachial (A) and common carotid artery (B) conductance at rest, during, and following water immersion. A two‐way ANOVA revealed main effects for artery and time (*P* < 0.01 and *P* < 0.05). *Significantly different from rest at *P* < 0.05. Data are mean ± SE.

A two‐way ANOVA revealed a significant effect for artery (*P* < 0.01), as brachial anterograde flow decreased significantly (*P* < 0.01), while the carotid remained unchanged (*P* = 0.93). Retrograde flow significantly increased in the brachial artery (*P* = 0.05), whereas carotid retrograde did not change (*P* = 0.56) (Table 1). Vascular conductance was also calculated and revealed significant main effects for artery (*P* < 0.01) and time (*P* < 0.05, Fig. 1).

**Table 1:** Vascular diameter and blood flows, during and after water immersion

Variable	Baseline	0–1 min	3–5 min	8–10 min	Rest
Carotid Artery Diameter (mm)	5.74 ± 0.49	5.99 ± 0.36^a^	6.05 ± 0.43^a^	6.12 ± 0.34^a^	5.92 ± 0.28
Carotid Artery Antegrade Flow (mL/min)	459 ± 163	486 ± 139	490 ± 132	503 ± 127	457 ± 99
Carotid Artery Retrograde Flow (mL/min)	−1 ± 3	0 ± 0	0 ± 0	0 ± 1	0 ± 1
Brachial Artery Diameter (mm)	3.94 ± 0.51	3.75 ± 0.47^a^	3.91 ± 0.52	3.96 ± 0.53	3.90 ± 0.59
Brachial Artery Antegrade Flow (mL/min)	101 ± 42	47 ± 22^a^	60 ± 25^a^	62 ± 24^a^	60 ± 21^a^
Brachial Artery Retrograde Flow (mL/min)	−3 ± 4	−10 ± 5^a^	−9 ± 6^a^	−12 ± 10^a^	−14 ± 10^a^

a Significantly different from rest at *P* < 0.05. Values are mean ± SD.

#### Diameters

**Figure 2:**
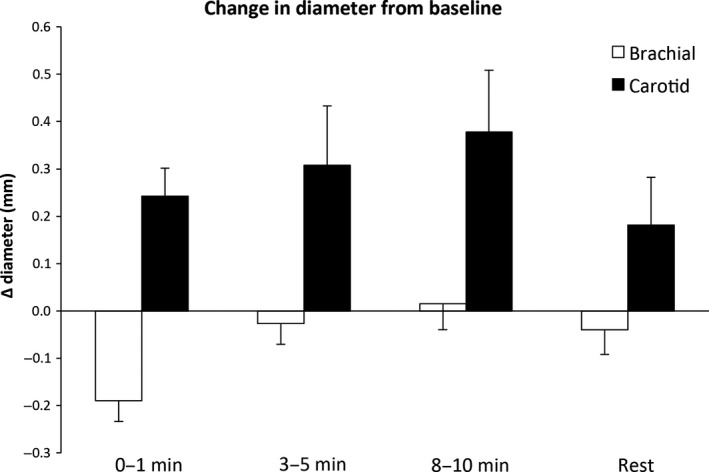
Change in diameter from baseline in the brachial (open bars) and common carotid artery (closed bars) during water immersion. A two‐way ANOVA revealed main effects for artery and time (*P* < 0.05 and *P* < 0.01) across the immersion protocol. Data are mean ± SE.

There was a significant change in brachial diameter throughout the immersion protocol (*P* < 0.01); however, further analysis revealed that diameter only decreased significantly at the 0–1 min time point and returned to baseline values for the remainder of the protocol. A significant increase in carotid artery diameter was observed at all time points during the immersion period (*P* < 0.01, Table 1), before returning to resting values once the tank was emptied. Finally, the change in diameter from baseline across the immersion protocol was significantly different between arteries (*P* < 0.05) (Fig. 2).

#### Shear rate

**Figure 3:**
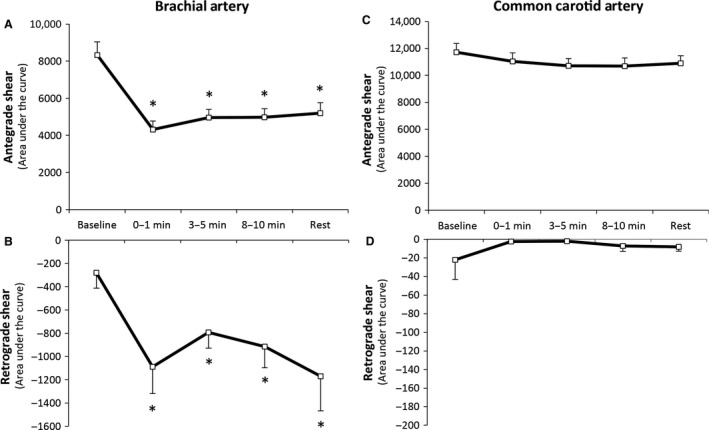
Brachial artery anterograde (A), retrograde (B), and common carotid anterograde (A) and retrograde (D) shear rates at rest, during, and following water immersion. A two‐way ANOVA revealed a significant difference between the arteries across the immersion protocol (*P* < 0.01). *Significantly different from rest at *P* < 0.05. Data are mean ± SE.

A significant difference in total shear rate area under the curve (SRAUC) was evident between the carotid and brachial arteries throughout the immersion protocol (*P* < 0.01, Fig. 3). Further analysis revealed that total SRAUC did not change in the carotid artery during immersion (*P* = 0.75), but decreased significantly in the brachial artery (*P* < 0.001). Anterograde shear decreased in the brachial artery (*P* < 0.001) while retrograde shear increased in the same artery (*P* < 0.05). No change in either anterograde (*P* = 0.74) or retrograde shear (*P* = 0.54) was evident in the carotid artery throughout the immersion protocol.

### Impact of water immersion on cardiovascular variables

A significant increase in MAP was evident during water immersion (baseline vs. 8–10 min; 80 ± 9 vs. 91 ± 12 mmHg, *P* < 0.01), along with CO (4.8 ± 0.7 vs. 5.1 ± 0.6 L/min, *P* < 0.05) and SV (62 ± 9 vs. 82 ± 5 mL, *P* < 0.01). Heart rate decreased significantly (78 ± 12 vs. 62 ± 10 bpm, *P* < 0.01). All variables returned to baseline levels once the tank was emptied.

## Discussion

This study is the first, to our knowledge, to illustrate that different vascular beds are exposed to distinct shear rate responses during exposure to the same systemic stimulus in resting humans. Total and anterograde shear rates in the brachial artery decreased significantly in response to water immersion, whereas there were no changes in either variable in the common carotid artery. Similarly, retrograde shear increased significantly in the brachial artery, while no change was apparent in the carotid. These distinct responses cannot be explained by differences in central hemodynamics, as recordings were made simultaneously. This highlights the differential nature of regional regulation of peripheral arterial beds in humans. Our findings indicate that adaptations to systemic stimuli, in particular changes in perfusion, may be vessel bed specific in humans, highlighting the need to measure multiple vascular sites.

The observation of decreased blood flow and shear rate in the brachial artery throughout the immersion period, in the face of elevations in blood pressure, is somewhat paradoxical as activation of cardiopulmonary or arterial baroreflexes might have been expected to induce peripheral vasodilation (Weisbrod et al. 2004). Forearm skin temperature does not change during immersion in 30°C water in our laboratory (Pugh et al. 2014), suggesting that it is unlikely that changes in brachial shear can be attributed to direct temperature‐driven vasoconstrictor effects. Our brachial artery and heart rate findings are, however, consistent with a diving reflex response to water immersion (Arborelius et al. 1972; Löllgen et al. 1981; Johansen et al. 1997; Foster and Sheel 2005). This reflex classically results in parasympathetic‐driven bradycardia and simultaneous sympathetic peripheral vasoconstriction, as observed in the present experiment. Although studies investigating the diving reflex in humans have classically utilized face immersion, our study excluded this form of stimulation, but did involve thoracic constriction. Previous reports suggest modulation of the diving reflex by pulmonary stretch receptors, whereby smaller lung volumes potentiate bradycardic responses (Andersson and Schagatay 1997) and lung volumes have well‐established impacts on peripheral sympathetic outflow (Seals et al. 1990, 1993; St. Croix et al. 1999). Our study is the first, we believe, to observe brachial vasoconstriction during water immersion, in the absence of facial immersion. Future experiments utilizing direct measurements of traffic along sympathetic nerves would help to further address mechanisms, although the study protocols would be difficult to undertake during microneurography.

The carotid artery blood flow responses we observed are consistent with our recent findings that water immersion at 30°C results in maintenance or small increases in intracranial blood flows (Carter et al. 2014) at rest. The explanation for the different responses we observed in the carotid and brachial arteries may relate to the fact that the brain has different intrinsic and extrinsic control mechanisms, as a stable cerebral blood supply is crucial for sustained functioning (Willie et al. 2014). There are a number of key regulators of cerebral blood flow, one being perfusion pressure. The ability of the brain to maintain a relatively constant blood supply in the face of changes in blood pressure is termed cerebral autoregulation. We observed no significant change in carotid flow, shear, or conductance in the face of increases in mean arterial pressure during the immersion protocol. However, carotid diameters increased significantly, indicating that regulation of carotid vasomotor activity was associated with the maintenance of flow and shear at near‐baseline levels. This observation reinforces recent suggestions that extracranial arteries may be involved in cerebral blood flow regulation (Willie et al. 2012, 2014). Interestingly, given that we induced responses similar to those of a diving reflex on the basis of the bradycardic and brachial vasoconstrictor findings, previous findings have also suggested that the diving reflex is associated with maintenance of carotid artery blood flows and redistribution of flows from the periphery to the cerebral circulations (Pan et al. 1997). This is in keeping with our current findings. However, it is also possible that increases in arterial CO_2_ levels observed during water immersion could be responsible for the dilation in the carotid artery (Carter et al. 2014), either by the direct effect of CO_2_ on vascular tone (Willie et al. 2012) or via a shear‐mediated mechanism (Carter et al. 2016). However, this seems unlikely as the increases in CO_2_ and carotid flow and shear were small (Carter et al. 2014).

Although this study benefits from a robust within‐subject design with simultaneous arterial measurements, it does have limitations. In this study we chose to use a water temperature of 30°C, as most public pools are maintained around this level. However, water temperature can impact on thermoregulation and cardiovascular function, therefore future studies should repeat this study design to characterize arterial flow and shear responses to a number of different graded water temperatures. We have previously shown that forearm skin temperature does not change during immersion to the RA in 30°C water, strongly suggesting that it does not activate thermoregulatory reflexes, however, core temperature was not measured in this study. Finally, we did not assess vascular flows and shears during an exercise stimulus, which should be addressed in future studies.

In conclusion, the findings of our study indicate that acute responses to a stimulus that induces systemic hemodynamic change at rest may be vessel bed specific in humans and depend upon a complex interplay between central factors, reflex changes, and localized responses to specific stimuli. These findings highlight the importance of examining multiple vascular territories in response to interventions that induce systemic changes in hemodynamics, such as different types of aquatic and land‐based exercise. Our study suggests that distinct regional vascular adaptations may occur in response to the same stimuli in humans.

## Conflict of Interest

None.
